# Prevalence, Intensity of Soil-Transmitted Helminths, and Factors Associated with Infection: Importance in Control Program with Ivermectin and Albendazole in Eastern Côte d'Ivoire

**DOI:** 10.1155/2019/7658594

**Published:** 2019-03-24

**Authors:** Agodio Loukouri, Aboulaye Méité, Olivier K. Kouadio, Norbert N. Djè, Gotré Trayé-Bi, Benjamin G. Koudou, Eliézer K. N'Goran

**Affiliations:** ^1^Université Félix Houphouët Boigny, 01 BP V 34, Abidjan 01, Côte d'Ivoire; ^2^Programme National de Lutte contre les Maladies Tropicales Négligées à Chimiothérapie Préventive, 06 BP 6394, Abidjan 06, Côte d'Ivoire; ^3^Centre Suisse de Recherche Scientifique, 01 BP 1303, Abidjan, Côte d'Ivoire; ^4^Université Nangui Abrogoua, 02 BP 801, Abidjan 02, Côte d'Ivoire

## Abstract

Evaluation of soil-transmitted helminths (STHs) and implementation of additional interventions are required in the region of a filariasis control program, given that antifilariasis drugs also have a beneficial effect on STHs. Thus, this study determines the extensive epidemiology of STHs to improve their successful control. Stool samples were analyzed using the Kato-Katz method. Chi-squared and Kruskal-Wallis tests were used to measure differences in infection rates and intensities, respectively, and logistic regression identified the risks of infection. The main intestinal helminths (*A. lumbricoides*, hookworm [*N. americanus*],* S. mansoni,* and* T. trichiura*) were found in the population. The overall prevalence of STHs was 19.5%. The prevalence of hookworm, the predominant species, ranged from 2% (n=6) to 28% (n=97). The overall prevalence of the other intestinal helminths was less than 6% (n=18). Intensity of hookworm was mostly light with a range from 1.6% (n=5) to 25.9% (n=90). However, the intensity of the species was significantly greater in Soribadougou compared to the other localities. Heavy infection was found in old children and adults but not in young children. Open defecation (OR=3.23, p≤0.05), dog/cat raising (OR=1.94, p≤0.05), farming (OR=14.10, p≤0.05), and irrigated culture (OR=3.23, p≤0.05) were positively associated with hookworm. It was observed that the participants missed the follow-up examinations due to trip (32.7%) or misunderstanding (15%) and lack of information (11.8%) of the purpose of the survey. Thus, to sustain the control of STHs, the MDA program should target the entire community and add education about the use of toilets, best practices of farming, and dog/cat raising.

## 1. Introduction

Soil-transmitted helminth (STH) infections and other neglected tropical diseases (NTDs) have often been omitted from public health priorities compared to the “big three diseases”: HIV/AIDS, tuberculosis, and malaria [1]. However, STHs are major public health problems in most tropical and subtropical countries [2]. Pathologies associated with STH infections may lead to acute illnesses, cognitive impairment, and sometimes long-term disability or early death [3]. In Africa, the three major STH infections, hookworm (*Necator americanus* and* Ancylostoma duodenale*), large roundworms (*Ascaris lumbricoides*), and whipworms (*Trichuris trichiura*) are often coendemic with* Schistosoma mansoni*, a trematode parasite [4]. Recently, NTD infections have received increased recognition [5]. Hence the World Health Organization (WHO) and its health partners are mobilizing resources and working together to achieve control and elimination where feasible.

The WHO recommended the use of anthelminthic drugs in preventive chemotherapy for the control of STH infections [6]. The anthelminthic drugs are usually directed at preschool-aged and school-aged children and women of childbearing age [7]. However, modelling studies on monitoring and evaluation of mass drug administration (MDA) have recently demonstrated that the adult population may even be the major reservoir for infection [8]. Hence, a key part of deciding whether community-wide MDA is appropriate should be an assessment of the level of infection in adults [9]. In addition, the long-term success of STH control programs requires additional interventions to the MDA [10] such as health education and improvement of water, sanitation, and hygiene (WASH) [11]. Health education promoted the use of sanitary latrines, prevention of soil pollution, and measures of personal prophylaxis such as wearing of protective footwear. The factors associated with STH infections vary considerably between geographical locations and are dependent on behaviors of the population and socioecological context [12]. In order to properly plan MDA and measure its impact on helminth infection, extensive knowledge of the epidemiology is crucial.

Côte d'Ivoire is a country located in Sub-Saharan Africa where STHs are endemic [13, 14]. In the eastern part of the country, the health districts of Akoupé and Abengourou are coendemic for lymphatic filariasis, onchocerciasis, and STHs. These areas were eligible for the lymphatic filariasis and onchocerciasis national MDA elimination program using ivermectin combined with albendazole. The treatment component of this program included all endemic districts to lymphatic filariasis in the country. Furthermore, the transmission assessment surveys (TAS) were done in the sentinel sites of Akoupé and Abengourou. An additional effect of this program is its potential impact on STHs, since the drugs used have a broad range anthelminthic activity [15]. The current study assesses the extensive epidemiology of STHs in sentinel sites of the filariasis control program. These data provide a sound basis to accurately estimate infection status and guide the choice of the intervention strategies to apply for STH control.

## 2. Materials and Methods

### 2.1. Study Design, Site and Participant Characteristic

**Figure 1:**
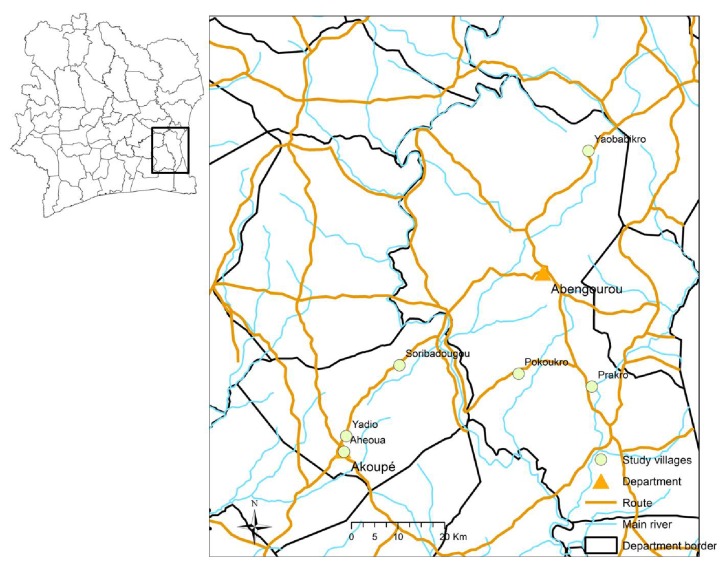
Map of study villages from the departments of Akoupé and Abengourou in eastern Côte d'Ivoire.

The villages from the health district of Akoupé and Abengourou were coendemic for lymphatic filariasis, onchocerciasis, and STH. These selected villages were eligible for community-wide MDA using ivermectin combined with albendazole. Preliminary surveys allow selecting localities with prevalence rates of* Wuchereria bancrofti *microfilaremia above 10% and* Onchocerca volvulus *nodule rates above 5%. Furthermore, no MDA with ivermectin for onchocerciasis elimination was performed for at least six months before the preliminary survey. Other criteria were acceptance of the survey and accessibility by car. Finally, six villages (Figure 1) were selected among the eligible villages by drawing lots.

These villages, located in the forest zone of eastern Côte d'Ivoire and characterized by warm and humid climate, are rural communities with only few socioeconomic differences. Ahéoua and Yadio are equipped with modern sanitation and drinkable water. Prakro has latrines and utilizes drinkable water. Pokoukro, Soribadougou and Yaobabikro possess latrines and use unsuitable water. In all six villages, agriculture is the main economic resource for the population.

The size of the population to be examined was calculated by considering that the test was bilateral, the test power should be 80%, and the Alpha risk threshold was 0.05. We ensure that the measured prevalence will be below specified limits, based on assumptions about the actual microfilaremia prevalence of* Wuchereria bancrofti* superior to 10%. Thus, a sample of at least 3200 participants was defined for all the sentinel sites. Given that the prevalence of STHs was estimated to be around 20%, at least, 1600 stools sample were required for the assessment of STH infections.

The study was carried out from 2014 (baseline survey) to 2017 (third follow-up survey). Individuals aged at least five years, without any acute diseases and for whom informed consent to participate in the surveys was provided, were included in the study. The follow-up surveys were all cross-sectional studies. A cross-sectional study had involved individuals present at the time point in the village without prior knowledge of their status regardless of the participation in the previous survey.

### 2.2. Field and Laboratory Procedures

The aim of the survey was explained to all participants and for minors to their parents. When the informed consent was obtained, a unique identification number was attributed and the participant received a labeled container that was to be filled with a fresh stool sample on the following day. Stool samples were collected and brought back to the laboratory where they were processed with Kato-Katz method for intestinal parasitic infection within the same day. Each stool sample was used to prepare two thick smears by using an amount of 41.7 mg of stool per slide with a specific plastic template [16]. In an interval of 30-60 min after the slides were made, they were examined by two experienced parasitologists. The quality control of the slides was carried out by a third experienced parasitologist. The quality control consisted of randomly selecting and reexamining 10% of the slides. In case of important disagreement between reading results (normal reading and quality control reading), the entire series of slides with differing results was reexamined.

### 2.3. Questionnaire Survey

In the year 2014, a questionnaire was administered to participants of parasitological study from three study sites (Soribadougou, Pokoukro, and Prakro) where the prevalence of hookworm was at least moderate. The purpose of the questionnaire was to identify risk factors of hookworm. Participants were selected by drawing lots. The questionnaire was related to the characteristics of drinking water, toilet disposal, hygiene practices, occupation, and pets raising. In the year 2017, a second questionnaire pertaining to the reasons that had prevented participation from follow-up surveys was done in one study site (Soribadougou). The other study sites did not receive the second questionnaire due to lack of financial resources.

### 2.4. Ethical Considerations

This study obtained clearance from the Comité d'Ethique et de la Recherche de Côte d'Ivoire (N/Réf: 001/MSHP/CNER-kp). The administrative and village authorities were informed about the procedures, benefits, and potential risks of the project. Informed consent from each participant to the study was obtained. Inclusion of minors (children under age) in the study required consent from parents as well as assent from each participant. The confidentiality of the data was preserved through a coding process.

### 2.5. Data Management and Analysis

All data were double-entered in Microsoft Excel file (2013 Microsoft Corporation) and crosschecked in EpiInfo version 3.5.3 (Center for Diseases Control and Prevention; Atlanta, United States of America). Participants were classified into six age groups: 5-10 years, 11-20 years, 21-30 years, 31-40 years, 41-50 years, and ≥ 51 years. The prevalence of infection was assessed by village and age group. Infection intensities were processed as log (eggs per gram of stool [epg] +1) to mimic skewness and null value in data. The mean infection intensities were computed by using geometric mean. Infection intensity of each species was stratified into three categories (light, moderate, and heavy) according to the WHO technical report [17].* A*.* lumbricoides* was defined between 1 epg to 4999 epg, 5000 epg to 49999 epg, and ≥50000 epg. The species* T. trichiura* was defined as follows: 1 epg to 999 epg, 1000 epg to 9999 epg, and ≥ 10000 epg.* Ancylostoma* spp. eggs were grouped between 1 epg and 1999 epg, 2000 epg and 3999 epg, and ≥4000 epg.* S*.* mansoni* was as follows: 1 epg to 99 epg, 100 epg to 399 epg, and ≥400 epg. Chi-squared test was used to indicate the difference in proportions between male and female participants. Difference in prevalence between villages was defined as nonoverlapping of confidence intervals. Due to nonnormal distribution of data, Kruskal-Wallis test was used to compare hookworm intensities. A significant outcome of the Kruskal-Wallis test led to a post hoc test to identify the group significantly different from the others. Statistical analyses were performed using STATA 12.00 software (Stata Corp., Texas 77845, USA). Binomial logistic regression was used to determine factors associated with hookworm infection. For this purpose, the association between hookworm infection was defined as the independent variable. Dependent variables were analyzed using univariate analysis and the strength of each association was measured with an odds ratio (OR). All variables with a minimum criterion of p value (p≤0.2) were specified and included in multivariate analysis at 95% confidence interval (CI). P value ≤0.05 was considered statistically significant.

## 3. Results and Discussion

### 3.1. Results

#### 3.1.1. Study Population

**Figure 2:**
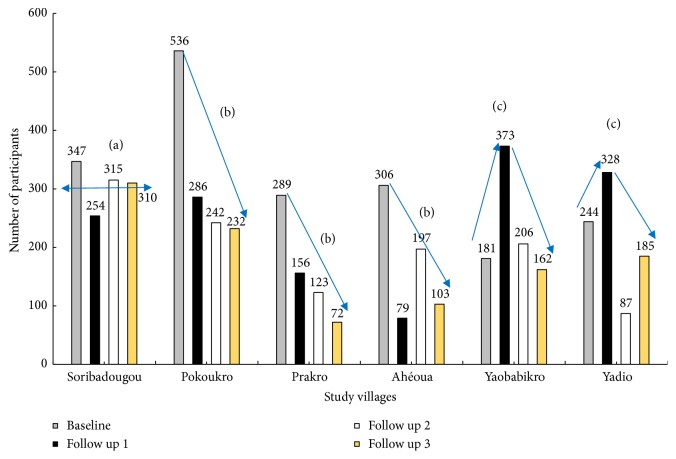
Participation over time for intestinal parasitological examinations in eastern settings of Côte d'Ivoire. (a) Relatively constant number of participants. (b) Significant decrease in the number of participants. (c) Bell-shaped evolution in the number of participants with a peak at first follow-up.

Overall, 1905 participants at baseline provided set of complete parasitological and demographic data distributed into six villages: Pokoukro (n=536), Soribadougou (n=347), Ahéoua (n=306), Prakro (n=289), Yadio (n=244), and Yaobabikro (n=181) (Figure 2). The highest proportion of participants was found with age group 11 to 20 years (33.6%, n=117) of Soribadougou and the lowest rate was recorded in age class of 40 to 49 years (1.4%, n=51) of the same village. Participation of females in the study was significantly higher than participation of males in Soribadougou (*χ*2=29.48, p≤0.05) and Yadio (*χ*2=14.3, p≤0.05). The mean age of individuals was 31, 36, 27, 28, 30, and 28 years, respectively, for Soribadougou, Yadio, Ahéoua, Pokoukro, Prakro, and Yaobabikro. Participation rates in parasitological examinations showed three types of evolution over time (Figure 2). The number of participants either remained relatively steady (Soribadougou), decreased considerably (Ahéoua, Pokoukro, and Prakro) with a reduction of around a half at first follow-up, or showed a bell-shaped evolution with the peak of participation reached during first follow-up survey (Yadio and Yaobabikro). Among the baseline population, 193 individuals from three villages, Soribadougou (n=70), Pokoukro (n=71), and Prakro (n=51) were interviewed about practices and behaviors related to hookworm infection. A total of 153 participants from the baseline survey in Soribadougou who had missed at least one follow-up survey were questioned on their motivation.

#### 3.1.2. Prevalence and Intensity Classes of Helminth Infections

**Figure 3:**
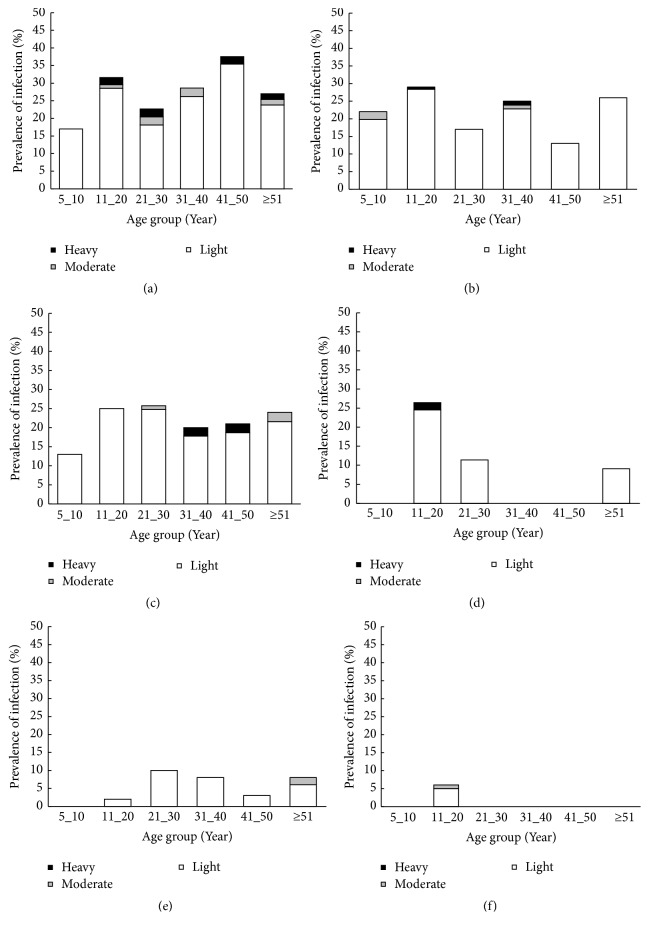
Prevalence and intensity categories of hookworm infection, stratified by age groups in eastern settings of Côte d'Ivoire. (a) Soribadougou. (b) Pokoukro. (c) Prakro. (d) Yaobabikro. (e) Yadio. (f) Ahéoua.

A total of four helminth species (*A. lumbricoides*, hookworm [*N. americanus*],* S. mansoni,* and* T. trichiura*) were observed. Overall, 21.3% of study population had helminth eggs detected in stool. The prevalence of STHs was estimated to be 19.5%. Hookworm was the predominant helminth species detected. The prevalence of this parasite ranged from 2.0% (Village of Ahéoua) to 28.0% (Village of Soribadougou) (Table 1). The other helminth species (*A. lumbricoides*,* T. trichiura,* and* S. mansoni*) had prevalence between 0% and 6.2%. The highest hookworm prevalence was observed in young age group (11 to 20 years) in Pokoukro (29%), young age group (11 to 20 years) and adult (21 to 30 years) in Prakro (25% and 27%), young age group (11 to 20 years) and adult (41-50 years) in Yaobabikro (34 and 35%), adults (21-30 years) in Yadio (10%), and adults (41-50 years) in Soribadougou (37.5%) (Figure 3). The lowest prevalence was detected in children aged 5-10 years in Soribadougou (17%), Yadio (0%), Yaobabikro (21%), and Prakro (13%) and with adults from 41 to 50 years in Pokoukro (13%). In Ahéoua, only young people aged 11 to 20 years were found positive for infection (6%). The coinfection of STH and* S. mansoni* and association of two STHs were found in proportions below 1%. Among the infected population, hookworm was mostly of light intensity (Table 1). People were significantly more infected in Soribadougou compared to the other villages (Pokoukro [*χ*2=4.44, p≤0.05], Prakro [*χ*2=3.06, p≤0.05], Yaobabikro [*χ*2=14.30, p≤0.05], Yadio [*χ*2=14.3, p≤0.05], and Ahéoua [*χ*2=14.30, p≤0.05]). Heavy infection was found in young and adults from Soribadougou, Pokoukro, and Prakro. No child (5-10 years) was heavily infected.

#### 3.1.3. Factors Associated with Hookworm and with Absence of Baseline Participants from Follow-Up Surveys

The proportion of households for one toilet was found to be 43.9, 33.7, 21.5, and 2.6% in the villages of Soribadougou, Pokoukro, Prakro, and Yaobabikro, respectively. The toilets include both latrines and the classic water closet. The latrines represented 100%, 91%, and 85% of the total number of toilets in Pokoukro, Prakro, and Yaobabikro, respectively. All toilets in Yadio (100%) and Ahéoua (100%), most toilets in Yaobabikro (65%) and Prakro (83%), and few toilets in Pokoukro (15%) and Soribadougou (8%) had roofing. The infection with hookworm was associated with open defecation (OR=3.23, p≤0.05) and dog/cat raising (OR=1.94, p≤0.05) in Soribadougou. Hookworm was linked to open defecation (OR=11.60, p≤0.05) and farming (OR=14.10, p≤0.05) in Prakro. Meanwhile, in the locality of Pokoukro, the activity of irrigated culture significantly predicts hookworm infection (OR=3.23, p≤0.05) (Table 2). The main reasons that prevented participants being in follow-up examinations were trip (32.7%), misunderstanding (15%), and lack of information (11.8%). Other contributing causes were presence of side effects (7.8%), fear of skin snip (7.8%), and the busy time of field work (7.7%). The refusal of blood collection (3.9%), the condition of obtaining previous result before being resampled (3.3%), fear of paying money (1.2%), and refusal of providing stool sample (1.0%) were of low importance. About 7.8% of interviewed individuals did not justify their absence.

## 4. Discussion

This study has determined the prevalence and intensity of STH infections, the factors associated with infection by hookworm, the most prevalent STH, and the causes that excluded participants from follow-up surveys in sentinel villages of a filariasis control program in eastern Côte d'Ivoire.

The overall prevalence of intestinal helminths (21.3%) and STHs (19.5%) found in this study is lower than most results obtained in studies of school-aged children across the country [18, 19]. However, our results were higher than those of few community surveys. The difference of prevalence rates reported may be due to the fact that evaluation of the current study took into account all community age groups including school-aged children, young, adults, and old people, whereas the previous studies were done in specific groups including socioprofessional and school-aged groups [19, 20]. Moreover, the other studies performed in communities of the country did not report the overall prevalence but focused on prevalence of specific parasite species [21, 22].

Most of the studies carried out in rural communities of Côte d'Ivoire also reported hookworm as the predominant STH [23–25]. These studies detected higher prevalence of hookworm (30% to 45%) compared to the present study (2% to 28%). However, the prevalence of hookworm in some study sites (Soribadougou [28.0%], Pokoukro [26.7%], and Prakro [25.3%]) is higher than the national average level of prevalence (17%) recorded among school-aged children few years ago [14]. As some studies carried out in the country and, elsewhere, school-aged children from more than 10 years have a high prevalence of hookworm infection [26]. Probably, this age group did not regularly receive anthelminthic treatment planned during the last decade by the health ministry. Another explanation may be the high level of reinfection of school-aged children with hookworm. The highest intensity (eggs per gram of stool) of hookworm was detected in adults. This result confirms the general epidemiology of the parasite [27] in the eastern area of Côte d'Ivoire. Therefore, hookworm deworming programs in rural eastern Côte d'Ivoire should target school-aged children as recommended by WHO and in addition also adults.

The presence of the other intestinal helminths (*A. lumbricoides*,* T. trichiura,* and* S. mansoni)* in the current study is in line with studies carry out in the country. The weak prevalence of* A. lumbricoides* and* T. trichiura* corroborated most of the results obtained in rural communities of Côte d'Ivoire [14, 28] and elsewhere in Africa [29, 30]. On the other hand, the species* S. mansoni* is known to be prevalent in the western region of Côte d'Ivoire [28].

The assessment of factors associated with hookworm indicated that open defecation and pet raising in Soribadougou and open defecation and practice of farming in Prakro were identified as predictors of hookworm infection [31, 32]. For the village of Pokoukro, residents who practice irrigated agriculture were at risk of hookworm infection. These outcomes are in line with previous findings on the epidemiology of hookworm infection [12, 33]. The high proportion of households without toilets may explain the behavior of large parts of the population to defecate in nature. The relatively steady participation of the population in Soribadougou suggests a deep understanding of the positive health impact and points to their constant availability and collaboration during the four years of the project. In the other study sites, human population was more collaborative at baseline or the first follow-up. The outcomes of the investigation of the main reasons for participants to continue to participate in parasitological examination can have an implication in the success of next steps of the MDA program. The primary cause mentioned for the lack of follow-up was that they were on trip during the parasitological surveys. This reason is two times more reported than the other causes of absence. A considerable percentage of the participants have declared that they did not understand the importance of the project for the wellbeing of the community. In addition, some people who already participated in previous surveys and agreed to be present during the follow-up surveys did not catch the information of time of the parasitological examination activity in their village. The truth or rumor about the appearance of side effects such as itching, swelling of the skin and healing delay or presence of wound due to skin snip were additional factors deterring some people from being resampled. Some individuals indicated that they had no time to spend at parasitological examination because they were very busy with the harvest season of their culture. In the current study, participants to the survey did not complain about blood collection or receipt of drug tablet after parasitological examination.

The full success of the MDA programs as the main strategy to control hookworm infection should take into account additional actions. These activities should be focused on regular use and maintenance of toilets by avoiding open defecation (Soribadougou and Prakro), education of farmers on good practice of culture (Prakro and Pokoukro), and best practices for raising of pets. Henceforth, for the next steps of the MDA program, more attention should be paid on mobilization of the population, including sharing details of the information with a particular emphasis on health importance and side effects of drugs and their management.

The study showed some limitations. The use of duplicate Kato-Katz alone as the diagnostic method offered more possibilities of missing STH eggs than multiplicate Kato-Katz or duplicate Kato-Katz associated with other diagnosis methods [34]. Another limit of the current study is the absence of baseline epidemiological data on preschool-aged children (0-4 years). Previous studies found this age group at risk of hookworm infection [35] that contributed to anemia and increased risk of morbidity among preschool-aged children [36]. On the other hand these children are more attracted to ground playing and are unconscious of risk factors of hookworm. They may play a role in reinfection of target population from control programs. The study did not screen all the study villages for the questionnaire survey.

The main intestinal helminths* S. mansoni*,* A. lumbricoides*,* T. trichiura,* and hookworm were found in the eastern settings of Côte d'Ivoire. Hookworm was the predominant species and the other helminths were detected in very low proportion. Hookworm infection was found in all age groups and the highest intensity was in adults. Factors associated with hookworm infection, depending on study sites, were either open defecation, practice of irrigated culture, or dog/cat raising. The MDA program should be addressed to all age categories and completed with health education on the use of improved toilets, best practice of culture, and dog/cat raising. Next steps of the MDA program should focus more on mobilization strategies of the community.

## Figures and Tables

**Table 1:** Prevalence and intensity of intestinal helminths in eastern settings of Côte d'Ivoire.

Intestinal helminths	Study villages					
	Ahéoua	Soribadougou	Pokoukro	Prakro	Yadio	Yaobabikro
*Schistosoma mansoni, n (*%)	9 (2.9)	5 (1.4)	13 (2.4)	18 (6.2)	1 (0.4)	1 (0.6)
*Ascaris lumbricoides, n (*%)	0 (0.0)	2 (0.6)	8 (1.5)	0 (0.0)	0 (0.0)	0 (0.0)
*Trichuris trichiura, n (*%)	2 (0.7)	4 (1.2)	2 (0.4)	0 (0.0)	2 (0.8)	1 (0.6)
*Hookworm, n (*%)	6(2.0)^c^	97(28.0)^a^	143(26.7)^a^	73(25.3)^a^	14(5.7)^b^	21(11.6)^b^
Geometric mean, epg	126.4^*α*^	313.1^*β*^	135.2^*α*^	162^*α*^	139.3^*α*^	141.4^*α*^
(95% CI)	(22.7-703.4)	(241.7-405.5)	(107.3-170.4)	(116.4-225.4)	(68.8-282.2)	(81.6-244.9)
Level of intensity						
No, n′ (%)	300 (98.0)	250 (72.0)	393 (73.3)	216 (74.8)	230 (94.3)	160 (88.4)
Light, n (%)	5 (1.6)	90 (25.9)	138 (25.7)	70 (24.3)	13 (5.3)	20 (11.0)
Moderate, n (%)	1 (0.4)	2 (0.6)	3 (0.6)	2 (0.6)	1 (0.4)	0 (0.0)
Heavy, n (%)	0 (0.0)	5 (1.5)	2 (0.4)	1 (0.3)	0 (0.0)	1 (0.6)
*Total, N (*%)	306 (100)	347 (100)	536 (100)	289 (100)	244 (100)	181 (100)

N: number of participants per study village, n: number of infected participants, n′: number of noninfected participants, epg: number of egg per gram of stool, %: percentage, ^a, b, c^: Chi-squared test significant ( p≤0.05), and ^*α*, *β*^: Kruskal-Wallis and independent t-tests significant ( p≤0.05).

**Table 2:** Factors associated with hookworm infection in eastern settings of Côte d'Ivoire.

Explanatory variable	Pokoukro					Prakro					Soribadougou				
		Univariate analysis		Multivariate analysis			Univariate analysis		Multivariate analysis			Univariate analysis		Multivariate analysis	
Sex	n (%)	OR	*p*	OR	*p*	n (%)	OR	*p*	OR	*p*	n (%)	OR	*p*	OR	*p*

Male	17 (*37*)	0.82	*>0.2*	NA	*NA*	28 (*87*)	5.09	*≤*0.2^*∗*^	4.59	*>0.05*	16 (*55*)	0.69	*>0.2*	NA	*NA*
Female	10 (*38*)					1 (*57*)					26 (*63*)				
School attending															
Yes	9 (*32*)	0.72	*>0.2*	NA	*NA*	29 (*80*)	2.07	*≤*0.2^*∗*^	3.68	*>0.05*	23 (*58*)	0.20	*>0.2*	NA	*NA*
No	18 (*41*)					10 (*66*)					19 (*61*)				
School level															
Primary school	25 (*39*)	0.49	*>0.2*	NA	*NA*	35 (*74*)	NA	*NA*	NA	*NA*	40 (*66*)	0.42	*>0.2*	NA	*NA*
College/high school	2 (*25*)					4 (*100*)					2 (*50*)				
Outside defecation															
Most of the time	20 (*47*)	3.41	*≤*0.2^*∗*^	2.25	*>0.05*	36 *(87*)	16.8	*≤*0.2^*∗*^	11.6	*≤*0.05^*∗∗*^	28 (*80*)	3.28	*≤*0.2^*∗*^	3.23	*≤*0.05^*∗∗*^
No/rarely	7 (*24*)					3 (*30*)					14 (*40*)				
Barefoot walking															
Most of the time	15 (*51*)	2.678	*≤*0.2^*∗*^	1.41	*>0.05*	23 (*76*)	1.03	*>0.2*	NA	*NA*	18 (*64*)	0.60	*>0.2*	NA	*NA*
No/rarely	12 (*28*)					16 (*76*)					24 (*57*)				
Occupation															
Yes	26 (*37*)	0.74	*>0.2*	NA	*NA*	39 (*78*)	NA	*NA*	NA	*NA*	34 (*65*)	1.54	*≤*0.2^*∗*^	0.95	*>0.05*
No	1 (*50*)					0 *(0*)					8 (*44*)				
Farming activity															
Yes	24 (*40*)	1.78	*>0.2*	NA	*NA*	31 (*86*)	5.42	*≤*0.2^*∗*^	14.1	*≤*0.05^*∗∗*^	32 (*65*)	1.37	*≤*0.2^*∗*^	0.51	*>0.05*
No	3 (*25*)					8 (*53*)					10 (*47*)				
Irrigated culture															
Yes	19 (*55*)	4.53	*≤*0.2^*∗*^	3.23	*≤*0.05^*∗∗*^	18 (*81*)	1.74	*>0.2*	NA	*NA*	29 (*65*)	1.31	*≤*0.2^*∗*^	0.66	*>0.05*
No	8 (*21*)					21 (*72*)					13 (*50*)				
Raising dog/cat															
Yes	19 (*42*)	1.644	*>0.2*	NA	*NA*	1 (*100*)	NA	*NA*	NA	*NA*	27 (*71*)	2.03	*≤*0.2^*∗*^	1.94	*≤*0.05^*∗∗*^
No	8 (*30*)					38 (*76*)					15 (46)				

n: number of positive individuals, NA: multivariate analysis not applicable, and OR: odd ratio. ^**∗**^: p value ≤ 0.2 ^*∗∗*^ . Statistically significant (p≤0.05).

## Data Availability

The data used to support the findings of this study are available from the corresponding author upon request.
